# Machine learning approach combined with causal relationship inferring unlocks the shared pathomechanism between COVID-19 and acute myocardial infarction

**DOI:** 10.3389/fmicb.2023.1153106

**Published:** 2023-03-29

**Authors:** Ying Liu, Shujing Zhou, Longbin Wang, Ming Xu, Xufeng Huang, Zhengrui Li, Andras Hajdu, Ling Zhang

**Affiliations:** ^1^Department of Cardiology, Sixth Medical Center, PLA General Hospital, Beijing, China; ^2^Department of Data Science and Visualization, Faculty of Informatics, University of Debrecen, Debrecen, Hungary; ^3^Faculty of Medicine, University of Debrecen, Debrecen, Hungary; ^4^Department of Clinical Veterinary Medicine, Huazhong Agricultural University, Wuhan, China; ^5^Department of Oral and Maxillofacial-Head and Neck Oncology, Shanghai Ninth People’s Hospital, Shanghai Jiao Tong University School of Medicine, Shanghai, China; ^6^College of Stomatology, Shanghai Jiao Tong University, Shanghai, China; ^7^National Center for Stomatology, Shanghai, China; ^8^National Clinical Research Center for Oral Diseases, Shanghai, China; ^9^Shanghai Key Laboratory of Stomatology, Shanghai, China; ^10^Shanghai Research Institute of Stomatology, Shanghai, China

**Keywords:** COVID-19, acute myocardial infarction, diagnostic biomarkers, machine learning, causal relationship, bioinformatics

## Abstract

**Background:**

Increasing evidence suggests that people with Coronavirus Disease 2019 (COVID-19) have a much higher prevalence of Acute Myocardial Infarction (AMI) than the general population. However, the underlying mechanism is not yet comprehended. Therefore, our study aims to explore the potential secret behind this complication.

**Materials and methods:**

The gene expression profiles of COVID-19 and AMI were acquired from the Gene Expression Omnibus (GEO) database. After identifying the differentially expressed genes (DEGs) shared by COVID-19 and AMI, we conducted a series of bioinformatics analytics to enhance our understanding of this issue.

**Results:**

Overall, 61 common DEGs were filtered out, based on which we established a powerful diagnostic predictor through 20 mainstream machine-learning algorithms, by utilizing which we could estimate if there is any risk in a specific COVID-19 patient to develop AMI. Moreover, we explored their shared implications of immunology. Most remarkably, through the Bayesian network, we inferred the causal relationships of the essential biological processes through which the underlying mechanism of co-pathogenesis between COVID-19 and AMI was identified.

**Conclusion:**

For the first time, the approach of causal relationship inferring was applied to analyzing shared pathomechanism between two relevant diseases, COVID-19 and AMI. Our findings showcase a novel mechanistic insight into COVID-19 and AMI, which may benefit future preventive, personalized, and precision medicine.

**Graphical abstract:**
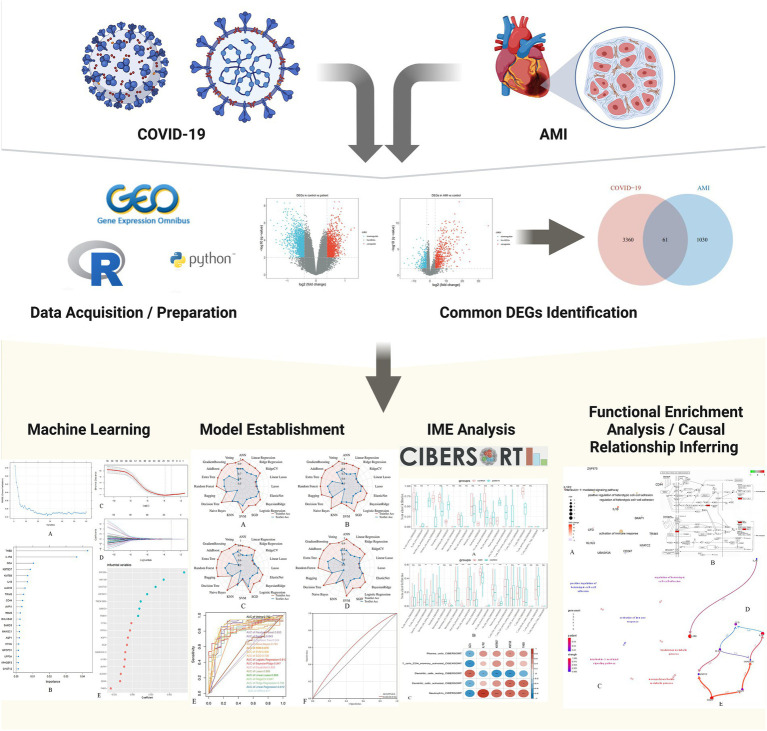

## Introduction

The emergence of the novel coronavirus 2019 (COVID-19) has triggered a global pandemic and posed unprecedented pressure on healthcare systems worldwide (Haldane et al., 2021; Lal et al., 2021). Today, it is well-realized that the severe acute respiratory syndrome coronavirus 2 (SARS-CoV-2) is the pathogen virus that causes COVID-19 and can further worsen it into severe lower respiratory tract infections in many mammals. Recently, many studies have pointed out that since the main target of the SARS-CoV-2 virus is the ACE receptor, a broadly existing surface receptor on diverse cell types across the whole human body, patients with COVID-19 infection are seemingly at a much higher risk of various life-threatening disease onset, such as cardiomyopathy, neuropathy, etc. (Kuderer et al., 2020; Lee et al., 2020; Rugge et al., 2020; Grivas et al., 2021; Li F. et al., 2021; Safiabadi Tali et al., 2021). However, although increasing evidence has shown that COVID-19 patients have an increased risk of sudden heart attacks, its connections with acute myocardial infarction (AMI) have not yet been identified to date. In fact, myocardial infarction, a heart muscle’s inability to receive enough oxygen and nutrients due to sudden blockage of the arteries, is one of the significant invisible hands of such heart diseases (Roth et al., 2017; Tsao et al., 2022). Statistically speaking, it is estimated that up to 8.3% of COVID-19-infected individuals may develop acute myocardial infarction, which is more than twice the incidence in the general population (Kumar et al., 2021; Toscano et al., 2021). Given the potential risk of AMI onset in the COVID-19-positive population, understanding such mechanisms is crucial. Hence, we investigated the shared pathomechanism between COVID-19 and AMI in the present study. We obtained gene expression profiles from the Gene Expression Omnibus (GEO). Having identified differentially expressed genes (DEGs) shared by COVID-19 and AMI, we performed a series of bioinformatics analyses to enhance the current understanding of this issue. We even developed a strong AMI diagnostic predictor for COVID-19-positive patients. From this end, we first attempted to identify the in-depth causal relationship between the two diseases based on their shared pathomechanism. As a result, our findings may provide further insight into future research and clinical practice regarding COVID-19 and AMI.

The general design of the present study is visualized in Graphical abstract.

## Materials and methods

### Data acquisition, preparation, and statistic management

GEO^1^ is an extensive gene expression database for various diseases that is freely available in the public domain. For COVID-19, we used the GSE164805 for analytics (Zhang et al., 2021). For AMI, we integrated GSE29111, GSE60993, GSE109048, GSE29532, GSE19339, GSE48060, GSE66360, and GSE97320 as a merged dataset (Silbiger et al., 2013; Suresh et al., 2014; Park et al., 2015; Muse et al., 2017; Gobbi et al., 2019). The normalization and calibration were done through the “normalizeBetweenArrays” function of the R package, “limma,” for both COVID-19 and AMI datasets (Supplementary Data S1). The analyses above were conducted by different R software packages and the integrative Python package “sklearn” (Pedregosa et al., 2011). If not specifically mentioned, the statistic test used in the analytics is the Wilcoxon rank sum test. Notably, within some figures, *, **, and *** may occur, indicative of a *p*-value < 0.05, 0.01, and 0.001, respectively.

### Identification of common DEGs between COVID-19 and AMI

In the present study, differential expression analysis was performed using the R package, “limma” (Ritchie et al., 2015). To avoid omission, DEGs were screened at a threshold of *p*-value < 0.05 and Log2 |fold change| > 1.00. After screening out the DEGs for COVID-19 and AMI, we crossed them to find common DEGs.

### Machine learning

The selection of feature genes to build the diagnostic predictor is crucial. In the present study, we first used the “RFE” algorithm to determine the ideal number of genes for formal modeling. Then we combined the linear algorithm, “LASSO,” with the non-linear algorithm, “Random Forest,” to narrow the list of potential genes of interest. As a result, the selected feature genes would be processed to construct the formal model (i.e., the AMI diagnostic predictor for COVID-19 patients).

For formal modeling, the whole AMI merged dataset was randomized and then separated into a training set and a test set at a ratio of 7.5:2.5. According to the “no-free-lunch” theorem, if one machine learning algorithm outperforms the others on a specific assessment, it should sacrifice certain points on the other assessment measurements (Wolpert and Macready, 1997). In short, nothing is perfect. However, through the exhaustive try-in of the mainstream machine learning algorithms and elucidation of different algorithms, we were able to choose the best one in general. Therefore, in the present study, 20 machine-learning algorithms, including Linear Regression, Ridge Regression, RidgeCV, Linear LASSO, LASSO, ElasticNet, BayesianRidge, Logistic Regression, SGD, SVM, KNN, Naive Bayes, Decision Tree, Bagging, Random Forest, Extra Tree, AdaBoost, GradientBoosting, Voting, and ANN, were compared and evaluated.

### Decision curve analysis

Usually, clinical models are absolutely and mathematically evaluated by the values of ROC-AUC, Accuracy, Precision, Recall, and F1-score without considering clinical outcomes. To overcome this disadvantage, Decision curve analysis (DCA) is used to compare the clinical benefits gained by employing different diagnostic predictors (Vickers and Elkin, 2006; Vickers et al., 2019). The more superior the curve localizes, the better prediction it outputs from the clinical aspect.

### Analysis of the immune microenvironment

CIBERSORT^2^ was used to assess the abundance of various infiltrating immune cells (Chen et al., 2018; Craven et al., 2021). Overall, 22 immune cell types were quantified. Correlation analysis between the immune cell types and GLS and SLC31A1 was done by the Pearson method. The visualization was created by the R package “ggplot2.”

### Functional enrichment analysis

Functional enrichment analysis included Gene Ontology (GO) terms and Kyoto Encyclopedia of Genes and Genomes (KEGG) pathways. The R package, “clusterprofiler” was used to carry out the functional enrichment analysis based on the common DEGs (Wu et al., 2021). The borderline criteria for selecting top enriched GO terms and KEGG pathways was with a significant adjusted *p*-value < 0.05.

### Causal relationship inferring

When studying gene expression profiling, inferring gene regulatory networks’ causality is crucial for investigating underlying molecular mechanisms. Herein, based on functional enrichment, we leveraged an advanced AI-essential R package, “CBNplot” to uncover the hidden secrets between COVID-19 and AMI (Sato et al., 2022).

## Results

### Identification of common DEGs between COVID-19 and AMI

**Figure 1:**
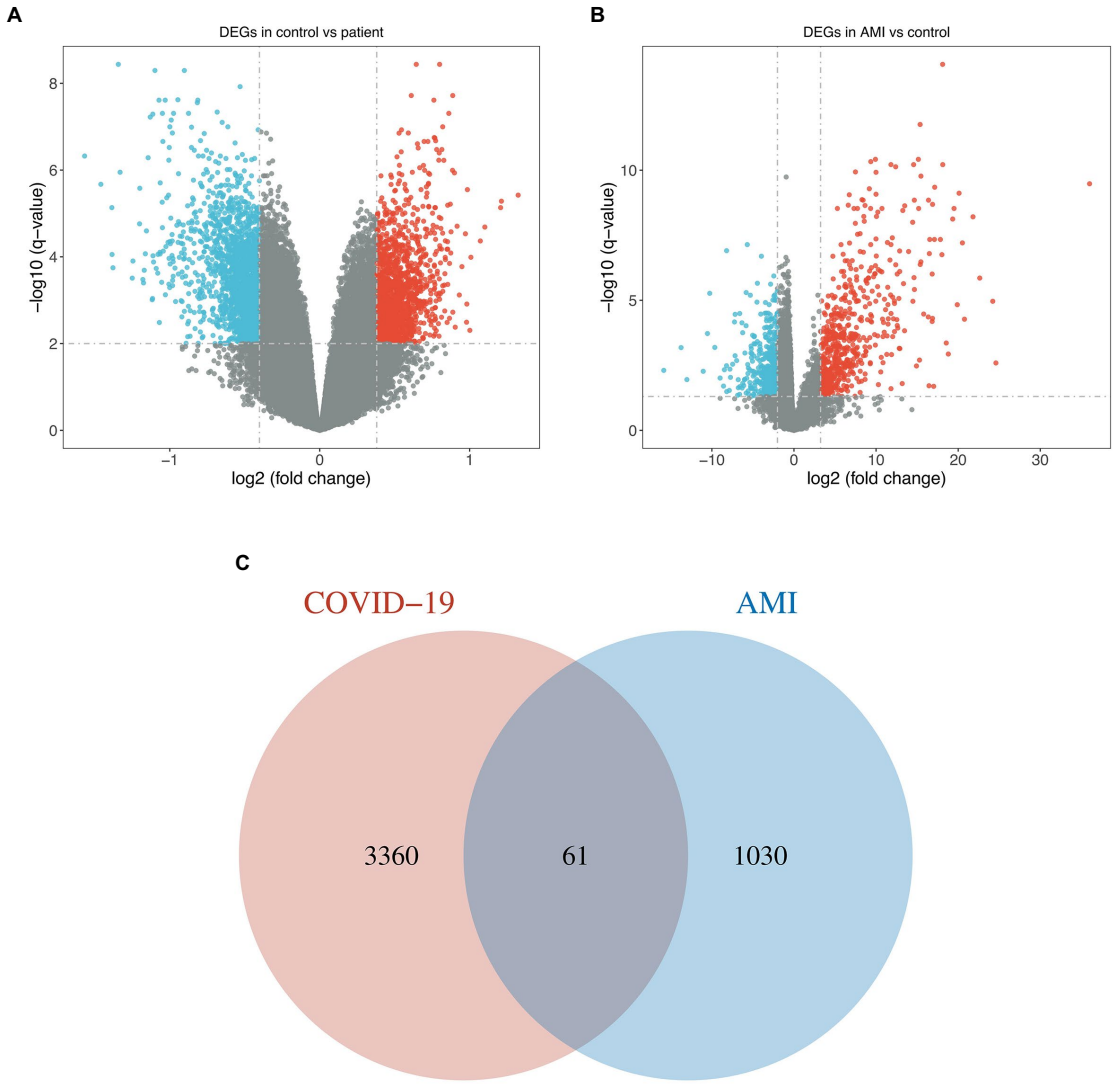
Identification of common DEGs between COVID-19 and AMI. **(A,B)** Volcano plots demonstrated the upregulated and downregulated DEGs of the COVID-19 dataset and merged AMI dataset. **(C)** Venn diagram shows the 61 common DEGs.

For the GSE164805 dataset, 3,421 DEGs were found, among which there were 1,527 genes upregulated and 1,894 genes downregulated (Figure 1A). For the merged AMI dataset, we identified 1,091 DEGs, including 483 upregulated genes and 608 downregulated genes (Figure 1B). By taking the intersection of DEGs of the GSE164805 dataset and the merged AMI dataset, there were 61 common DEGs found, which were visualized by Venn diagrams (Figure 1C; Supplementary Figure S1).

### Pre-modeling: Integrative approach for feature genes selection

**Figure 2:**
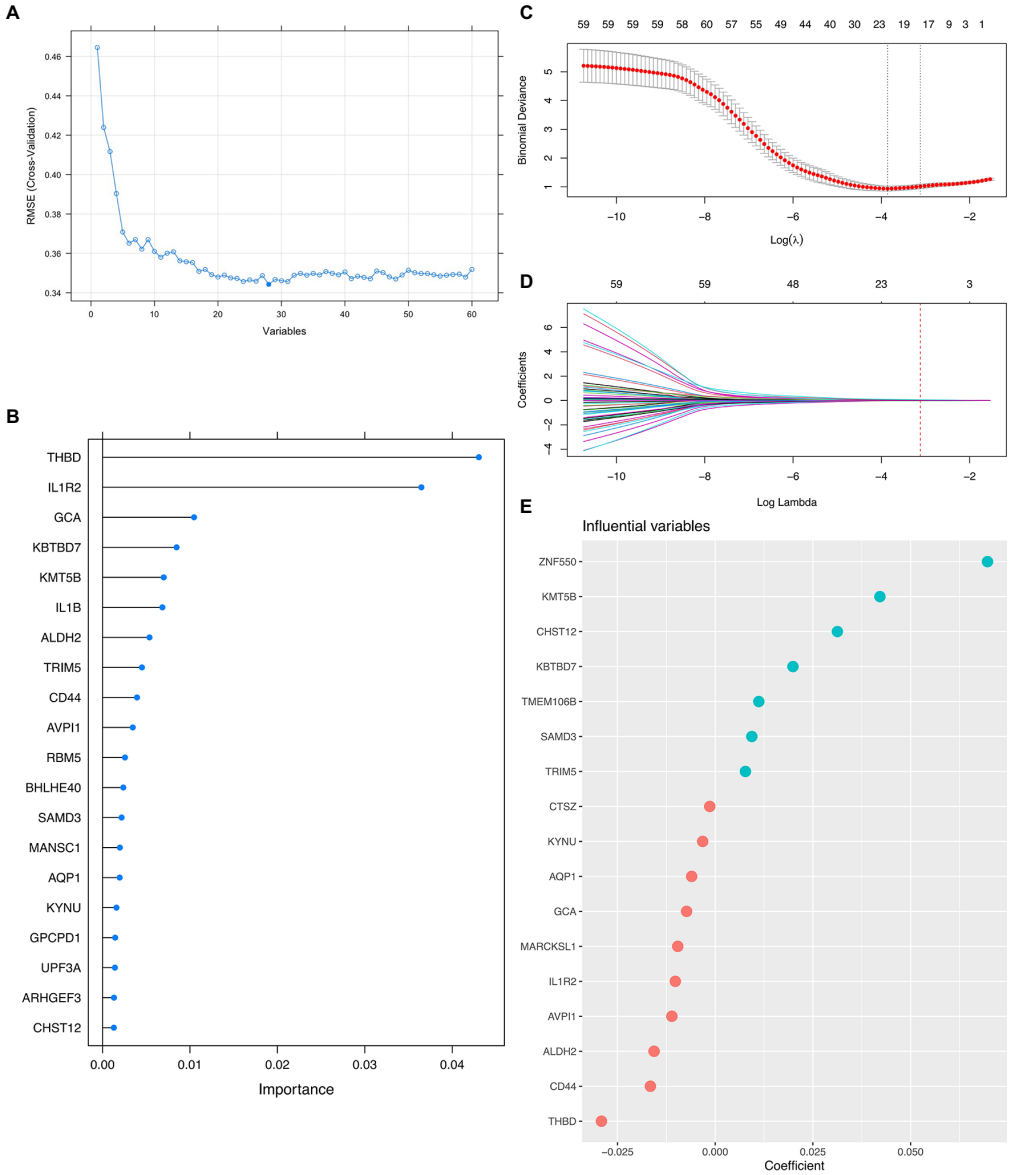
Integrative Approach for Feature Genes Selection. **(A)** Scree plot demonstrating the fluctuation of the RMSE value against the number of variants (i.e., feature genes) involved in formal modeling. **(B)** Lollipop plot shows each top 20 feature gene’s importance by the Random Forest algorithm. **(C,D)** Dot plots and curves demonstrate the binormal deviance changes and coefficient allocation process against the value of Log Lambda, respectively. **(E)** Bubble plot showing the importance of each top 20 feature genes by LASSO.

Since the mathematical relationship between the predictors and the outcome was unknown, we combined both linear (i.e., LASSO) and non-linear (i.e., Random Forest) methods to filter out the most promising genes for formal modeling after determining the ideal number of genes that the RFE algorithm should use. As a result, 5 genes were believed to be the best option since, after this point, the RMSE-value fluctuated on a tiny scale, suggesting only little changes in the predictive powerfulness occurred (Figure 2A). On the other hand, the Random Forest algorithm ranked the importance of each top 20 genes, in which THBD, IL1R2, GCA, KBTBD7, and KMT5B were the uppermost (Figure 2B). Furthermore, the LASSO narrowed down the binormal deviance to the minimum (Figure 2C) and allocated a coefficient to each gene (Figure 2D), also showing the top 20 most weighted genes (Figure 2E). After that, we selected the overlapping genes from the top 20 genes given by the Random Forest algorithm and the LASSO for formal modeling.

### Formal modeling: Establishing an AMI diagnostic predictor for COVID-19 patients

**Figure 3:**
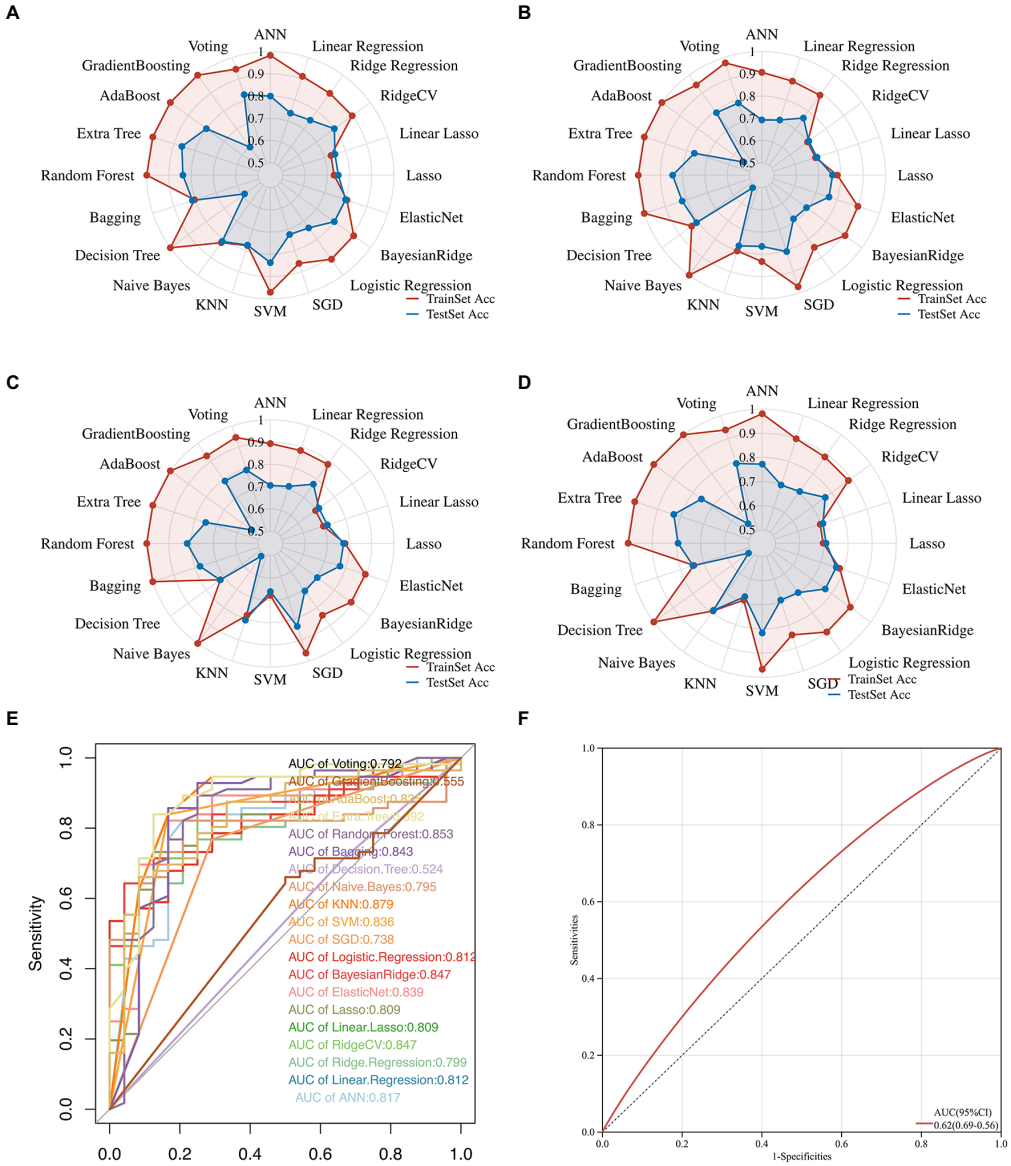
Multifaceted evaluation of 20 mainstream machine-learning diagnostic predictors. **(A–D)** The Radar plot demonstrates accuracy, recall, and F1-score measurement in the train and test sets, respectively. **(E)** Receiver Operative Curve (ROC), in which each predictor’s Area Under Curve (AUC) value was compared. Generally, an AUC value over 0.7 was considered a good predictive performance. **(F)** ROC of the cardiac troponin.

Herein, we attempted 20 different machine learning methods currently under service in the field so that the data, in terms of the predictive performance and property, could be fit as optimally as possible. Consequently, Extra Tree exerted the maximum performance regarding the Accuracy, Precision, Recall, and F1-score, followed immediately by Random Forest, and then SVM (Figures 3A–D; Supplementary Figure S2). In addition, the value of ROC-AUC of Extra Tree was the highest among the candidates, up to 0.892 in the test dataset (Figure 3E). Meanwhile, cardiac troponin, a gold standard biomarker for AMI, only possessed a ROC-AUC value of 0.62. Therefore, it was deemed that the Extra Tree algorithm was much superior (Figure 3F).

### Exploration of the feature genes’ implications in immunology

**Figure 4:**
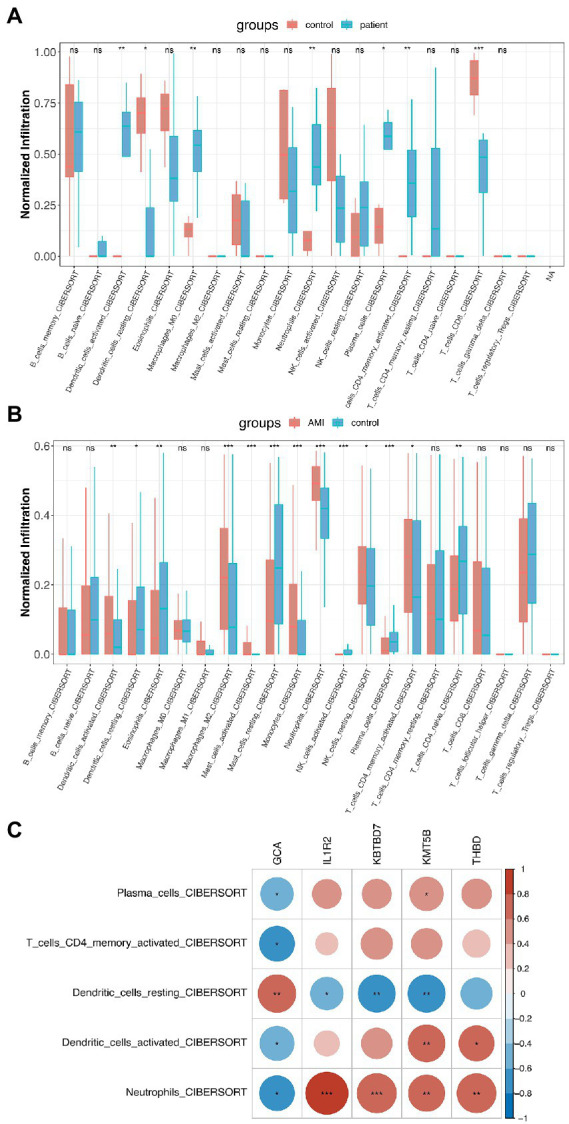
Immunological implications of the feature genes. **(A)** Comparison of infiltrating immune cells in patients who were COVID-19-positive and-negative. **(B)** Comparison of infiltrating immune cells in patients with AMI and healthy controls. **(C)** Correlation analysis of feature genes and the 5 shared differentially regulated immune cells.

With the help of the CIBERSORT platform, it was observed that in COVID-19-positive patients, Plasma Cells, Activated CD4 Memory T Cells, CD8 T Cells, both Activated and Resting Dendritic Cells, M0 Macrophages, and Neutrophils were statistically different from that in COVID-19-negative patients (Figure 4A). Interestingly, besides Resting Dendritic Cells, CD8 T Cells were less abundant in COVID-19-positive patients. For AMI, Plasma Cells, Activated CD4 Memory T Cells, CD4 Naïve Cells, both Activated and Resting Dendritic Cells, both Activated and Resting Mast Cells, both Activated and Resting NK Cells, Macrophage M2, Eosinophils, and Neutrophils were statistically different from that in healthy controls (Figure 4B). Then, the 5 shared differentially regulated immune cells (i.e., Plasma Cells, Activated CD4 Memory T Cells, both Activated and Resting Dendritic Cells, and Neutrophils) were screened out. They underwent a correlation analysis with the feature genes (Figure 4C). Subsequently, it was found that the GCA gene was statistically associated with all 5 shared differentially regulated immune cells, and Neutrophils were statistically associated with all the feature genes. However, except for Resting Dendritic Cells, the GCA gene was negatively correlated with the other shared differentially regulated immune cells, hindering it might serve as an inhibitor in the immune system activation in the shared pathomechanism of COVID-19 and AMI.

### Functional enrichment analysis and causal relationship inferring

**Figure 5:**
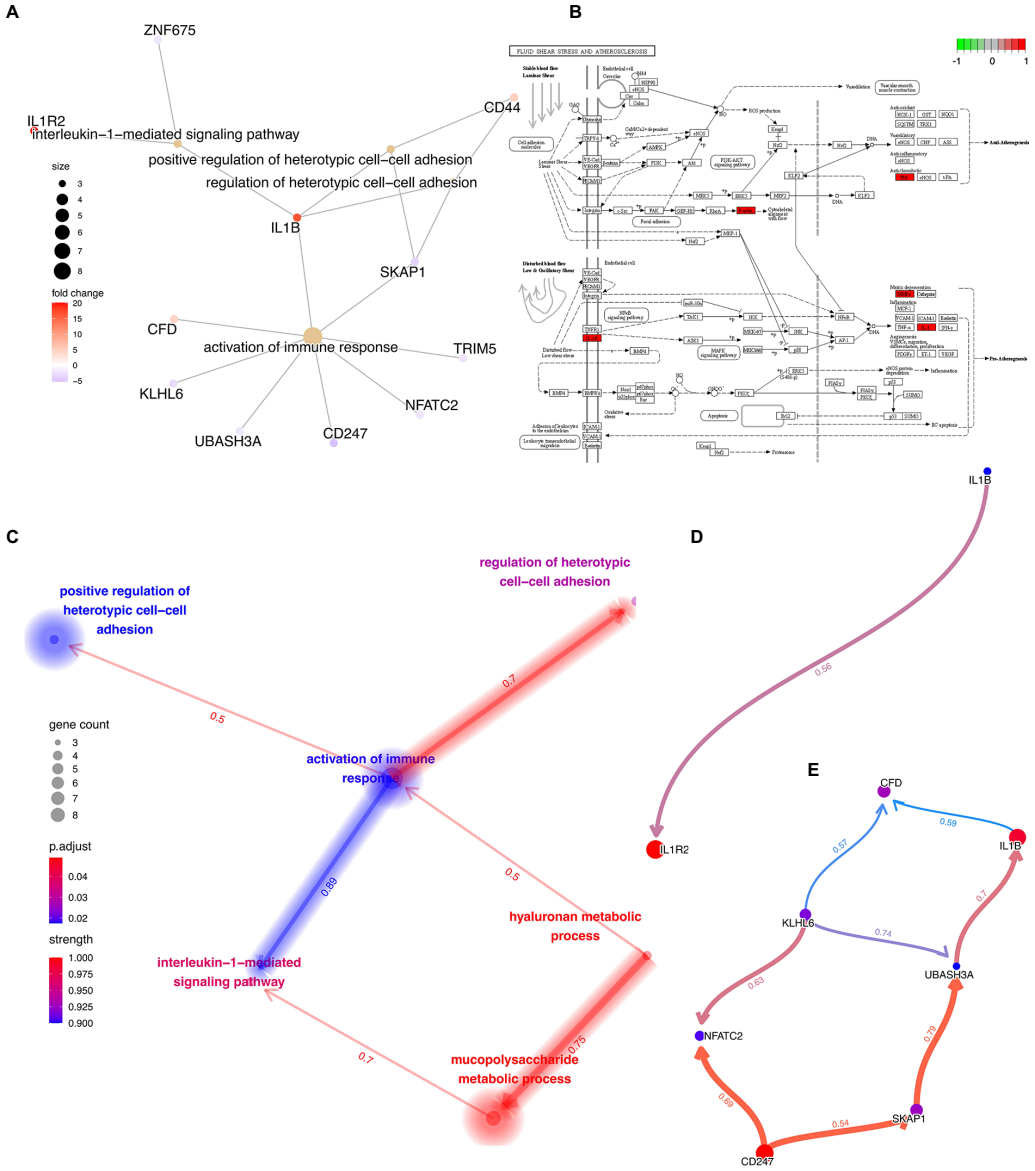
Functional Enrichment Analysis and Causal Relationship Inferring. **(A)** Interactive network demonstrating the interactions between the feature genes and the enriched GO terms and KEGG pathways. **(B)** Detailed KEGG pathway, “Fluid shear stress and atherosclerosis,” activated genes are marked in red. **(C)** Interactive network demonstrating the interactions between the different GO terms and KEGG pathways with directions. The directions of the arrows indicate the causal relationship. **(D,E)** Complex causal relationship inferred within the enriched GO terms, “activation of immune response” and “interleukin-1-mediated signaling pathway.”

First, a traditional functional enrichment analysis was performed, in which we identified 6 statistically significant GO terms and 1 KEGG pathway. The enriched GO terms included “hyaluronan metabolic process,” “interleukin-1-mediated signaling pathway,” “regulation of heterotypic cell–cell adhesion,” “activation of immune response,” and “positive regulation of heterotypic cell–cell adhesion” (Figure 5A). The KEGG pathway was “Fluid shear stress and atherosclerosis” from which the more precise subpathway was “Atherogenesis” (Figure 5B). Then, we employed the R package “CBNplot” to infer the causal relationships between them, the results of which could be verified through probabilistic inferring and classification according to the explanation of Sato et al. (Figure 5C). Herein, we found that “activation of immune response” served as a core within the interactive network and exhibited the most robust causal relationship with the “interleukin-1-mediated signaling pathway,” indicative of their significance in the co-pathogenesis of COVID-19 and AMI. The direction was from “activation of immune response” to “interleukin-1-mediated signaling pathway.” The details are visualized in Figures 5D,E. By observing the genes involved and the directions of the vectors, it was thought that IL1B seemingly played the most critical role.

## Discussion

Cardiovascular disease is an essential cause of the global burden of death, far exceeding cancer. Most of these deaths were due to acute myocardial infarction (AMI; Roth et al., 2017; Tsao et al., 2022). At the same time, with the gradual severity of the epidemic, like similar epidemic diseases, COVID-19 has also brought more adverse complications (Anastasiou et al., 2012; Del Sole et al., 2020; Li et al., 2020, 2022; Lippi et al., 2020; Li X. et al., 2021; Ramphul et al., 2021a,b; Chai et al., 2022). Accumulating evidence shows that the prevalence of AMI in COVID-19 patients is much higher than that in the uninfected population (Kumar et al., 2021; Toscano et al., 2021). This compels us to look for the mechanisms underlying the interaction between these two diseases and to explore the potential behind this complication. As a result, we found potential drug targets for COVID-19 and its related AMI, leaving a theoretical basis for diagnosing and treating related diseases.

We obtained gene expression data for COVID-19 and AMI from the GEO database. On this basis, 61 shared differentially expressed genes (DEGs) were screened out, and a series of systematic and bioinformatics analyzes were performed. We also developed a robust predictor from 20 mainstream machine-learning algorithms to estimate the risk of AMI in COVID-19 patients. Most notably, we infer causal relationships among the most important biological processes through Bayesian networks. Through these processes, we identified mechanisms underlying the co-pathogenesis of COVID-19 and AMI.

Among all DEGs, THBD, IL1R2, GCA, KBTBD7, and KMT5B were found to be most important in the LASSO and the Random Forest algorithm. THBD and its encoded thrombomodulin play an important role in forming venous thrombosis and vascular inflammation (Ireland et al., 1997; Doggen et al., 1998; Anastasiou et al., 2012). They also have unique roles in other non-thrombotic cardiovascular diseases such as AMI. Zhao et al. have reasoned that IL1R2 is a common marker gene of myocardial infarction, especially closely related to immune infiltration in AMI patients (Zhao et al., 2020). GCA drives coronary ischemia and promotes the development of immune cell arterial inflammation (Akiyama et al., 2021). KBTBD7 is now one of the most promising targets in the AMI. The researchers targeted and regulated KBTBD7 through various measures to inhibit inflammation, cardiac dysfunction, and maladaptive remodeling after myocardial infarction with weak downstream p28 and NF-κB signaling. One example of such a complex network is the work of Yang et al. in 2018, in which they found that MiR-21 suppressed AMI by targeting KBTBD7 and controlling p38 and NF-κB signaling pathways (Qian et al., 2011; Liu et al., 2015; Yang et al., 2015). Interestingly, KMT5B is considered in traditional biological science to be a key gene regulating stem cell and neurological development (Chen et al., 2022; Hulen et al., 2022). Only recently has it been discovered that it plays a vital role in vascular endothelial cell inflammation and angiogenesis (Guo et al., 2015). The above-mentioned key DEGs reveal the disease characteristics of AMI caused by COVID-19 to a certain extent. They may prove that COVID-19 can induce thrombosis and even AMI formation through vascular inflammation triggered by cell inflammation.

Inspired by the latest advancement in artificial intelligence, we traversed 20 mainstream machine learning algorithms to fit the data and improve performance (Wolpert and Macready, 1997; Xie et al., 2023). We found that Extra Tree had the highest predictive performance. For further validation, we compared it to cardiac troponin, a recognized gold standard biomarker for AMI. Interestingly, we found that the predictive power of Extra Tree (AUC = 0.892) was much higher than that of cardiac markers such as cardiac troponin (AUC = 0.62). This indicates that the Extra Tree predictor has a very high accuracy for AMI diagnosis in COVID-19 patients, posing a challenge to traditional biomarkers and inspiring us to mine out more potential but promising novel biomarkers in the future.

In addition, we explored the immunological association between these two diseases. We found that the highly active immune cells were nearly identical in both diseases. Plasma Cells, Activated CD4 Memory T Cells, Activated and Resting Dendritic Cells, and Neutrophils are key secretory cells of cellular immune factors. It is believed that the excessive inflammatory response and cytokine storm induced by the virus can lead to myocardial injury, which may be one of the key factors in the occurrence of AMI after COVID-19.

The highly active “activated immune response” and “interleukin-1-mediated signaling pathway” verified our previous findings to a certain extent. Both DEGs and immunoassays confirmed that the disease of COVID-19 and AMI is immune-focused and can even be specific to the activation of IL1-related immune pathways. In the post-coronavirus epidemic era or the long coronavirus era, we can start from this immune pathway to explore the key damage pathways of COVID-19 in the circulatory system and then find new preventive measures.

A limitation of this study is that we could not model COVID-19 and AMI disease in animals due to the level of laboratory safety required by COVID-19. However, as an exploratory pioneer study, for the first time in history, we have applied a causal inference approach to studying the shared pathogenesis of COVID-19 and AMI. Our findings demonstrate novel mechanistic insights into COVID-19 and AMI that may aid future prevention, personalized and precision medicine.

## Data availability statement

The original contributions presented in the study are included in the article/Supplementary material, further inquiries can be directed to the corresponding authors.

## Author contributions

YL and SZ: conceptualization and methodology. YL, SZ, LW, MX, XH, and AH: software and validation. YL, SZ, AH, and ZL: formal analysis and writing - original draft preparation. LW, MX, and XH: data curation. AH, ZL, and LZ: supervision, funding acquisition and writing - review and editing. SZ, XH, AH, and ZL: visualization. XH and LZ: project administration. All authors have read and agreed to the published version of the manuscript.

## Funding

The work was supported by the National Natural Science Foundation of China (81771127), the Seed Foundation of the Ninth People’s Hospital, Shanghai Jiao Tong University School of Medicine (JYZZ196), and by the project TKP2021-NKTA-34, implemented with the support provided by the National Research, Development, and Innovation Fund of Hungary under the TKP2021-NKTA funding scheme.

## Conflict of interest

The authors declare that the research was conducted in the absence of any commercial or financial relationships that could be construed as a potential conflict of interest.

## Publisher’s note

All claims expressed in this article are solely those of the authors and do not necessarily represent those of their affiliated organizations, or those of the publisher, the editors and the reviewers. Any product that may be evaluated in this article, or claim that may be made by its manufacturer, is not guaranteed or endorsed by the publisher.

## Footnotes

^1^ www.ncbi.nlm.nih.gov/geo

^2^ https://cibersort.stanford.edu/
