# Supplementary figures and images for: Machine learning approach combined with causal relationship inferring unlocks the shared pathomechanism between COVID-19 and acute myocardial infarction
Source: Front Microbiol. 2023 Mar 29;14:1153106. doi: 10.3389/fmicb.2023.1153106 (PMC10090501; doi:10.3389/fmicb.2023.1153106)

**log transform**

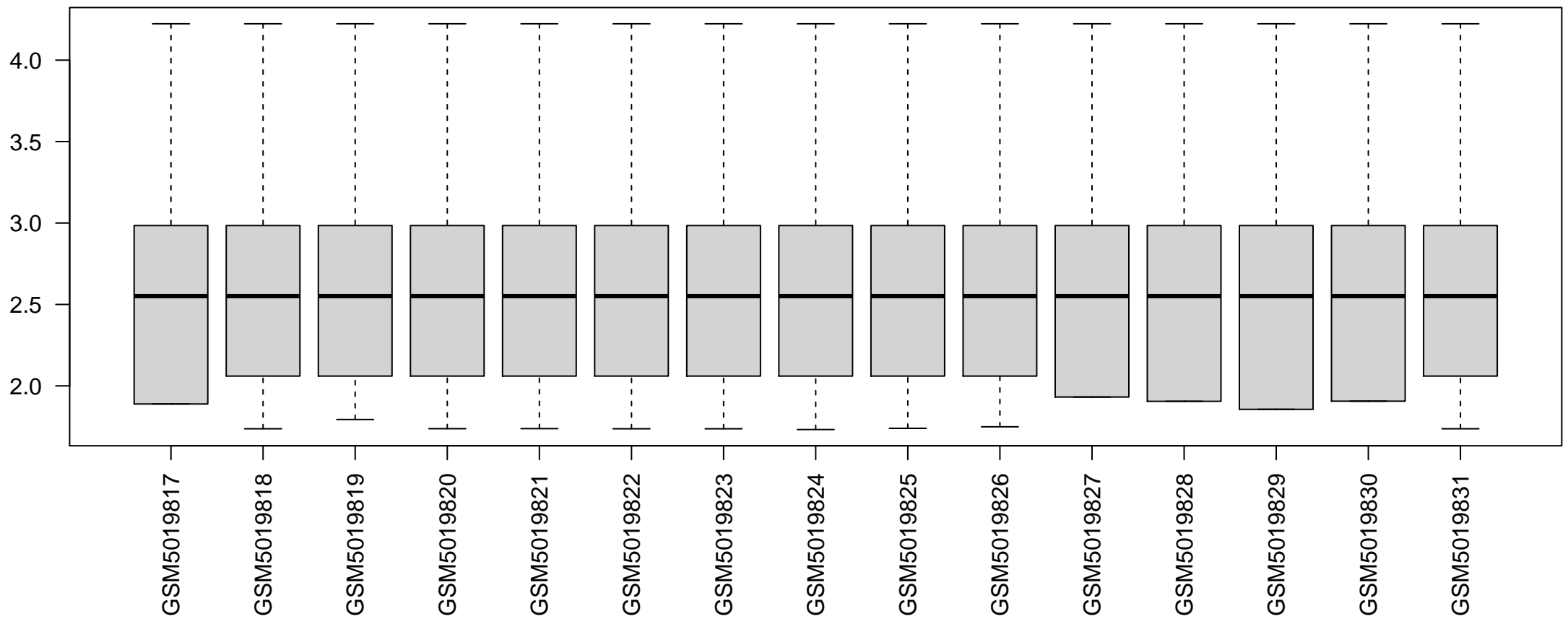

**remove array difference**

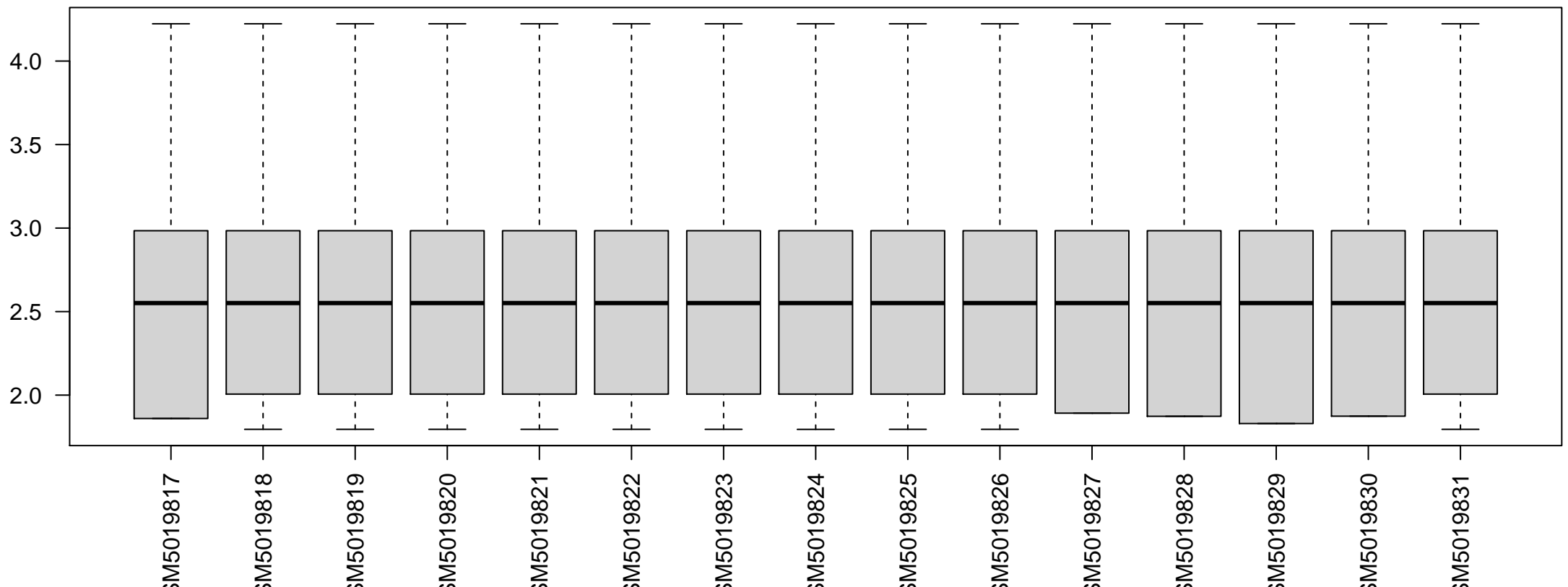

Supplement: Supplementary file 2 [file Image_1.PDF]

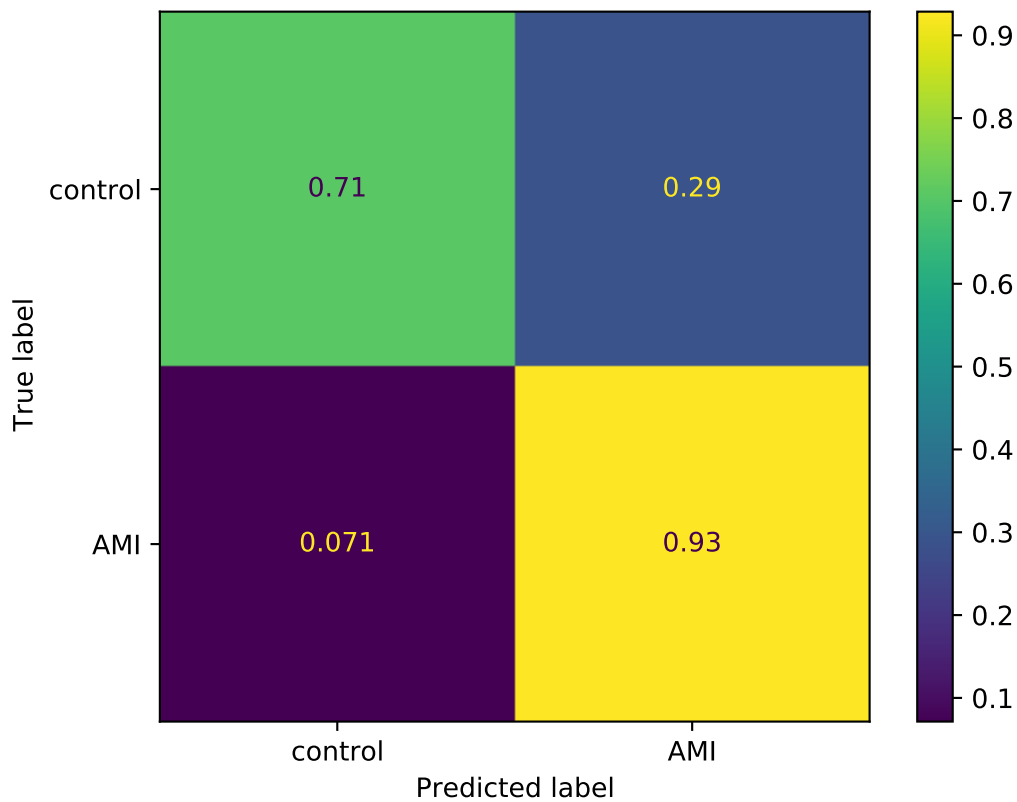

Supplement: Supplementary file 3 [file Image_2.PDF]
